# The price of protection: a defensive endosymbiont impairs nymph growth in the bird cherry‐oat aphid, *Rhopalosiphum padi*

**DOI:** 10.1111/1744-7917.12606

**Published:** 2018-07-25

**Authors:** Daniel J. Leybourne, Jorunn I. B. Bos, Tracy A. Valentine, Alison J. Karley

**Affiliations:** ^1^ Division of Plant Sciences, School of Life Sciences University of Dundee Dundee UK; ^2^ Cell and Molecular Sciences the James Hutton Institute Invergowrie Dundee UK; ^3^ Ecological Sciences the James Hutton Institute Invergowrie Dundee UK

**Keywords:** cereal aphid, *Hamiltonella defensa*, *Hordeum spontaneum*, *Hordeum vulgare*, symbiosis

## Abstract

Bacterial endosymbionts have enabled aphids to adapt to a range of stressors, but their effects in many aphid species remain to be established. The bird cherry‐oat aphid, *Rhopalosiphum padi* (Linnaeus), is an important pest of cereals worldwide and has been reported to form symbiotic associations with *Serratia symbiotica* and *Sitobion miscanthi* L‐type symbiont endobacteria, although the resulting aphid phenotype has not been described. This study presents the first report of *R. padi* infection with the facultative bacterial endosymbiont *Hamiltonella defensa*. Individuals of *R. padi* were sampled from populations in Eastern Scotland, UK, and shown to represent seven *R. padi* genotypes based on the size of polymorphic microsatellite markers; two of these genotypes harbored *H. defensa*. In parasitism assays, survival of *H. defensa*‐infected nymphs following attack by the parasitoid wasp *Aphidius colemani* (Viereck) was 5 fold higher than for uninfected nymphs. Aphid genotype was a major determinant of aphid performance on two *Hordeum* species, a modern cultivar of barley *H. vulgare* and a wild relative *H. spontaneum*, although aphids infected with *H. defensa* showed 16% lower nymph mass gain on the partially resistant wild relative compared with uninfected individuals. These findings suggest that deploying resistance traits in barley will favor the fittest *R. padi* genotypes, but symbiont‐infected individuals will be favored when parasitoids are abundant, although these aphids will not achieve optimal performance on a poor quality host plant.

## Introduction

Aphids form a diverse range of associations with endosymbiotic bacteria, ranging from obligatory to facultative and beneficial to parasitic. The primary aphid endosymbiont, *Buchnera aphidicola*, provides nutritional supplementation to the aphid diet (Sasaki *et al*., 1991; Douglas & Prosser, 1992). Additional coobligatory symbioses with *B. aphidiciola* have been described in other aphid species, including cosymbiosis with *Wolbachia* sp. in the banana aphid, *Pentalonia nigronervosa* (Coquerel) (De Clerck *et al*., 2015), and *Serratia symbiotica* in *Cinara* species (Meseguer *et al*., 2017). The most frequently detected facultative endosymbionts of aphids are *Hamiltonella defensa*, *Regiella insecticola*, *S. symbiotica*, *Rickettsia* sp., *Ricketsiella* sp., *Spiroplasma* sp., the *Pea Aphid X‐type Symbiont* (*PAXS*) and *Wolbachia* sp. (Sandström *et al*., 2001; Oliver *et al*., 2003; Oliver *et al*., 2006; Degnan & Moran, 2008b; Guay *et al*., 2009; Oliver *et al*., 2010; Tsuchida *et al*., 2010; Łukasik *et al*., 2013b; De Clerck *et al*., 2014). A concise review of endosymbiont occurrence in aphid populations (Zytynska & Weisser, 2016) found that the facultative endosymbionts *S. symbiotica* and *Wolbachia* infected the highest proportion of the aphid species assessed (47% and 43%, respectively). Occasional associations have also been reported with *Arsenophonus* sp. (Jousselin *et al*., 2013; Wagner *et al*., 2015), infecting 7% of aphid species tested (Zytynska & Weisser, 2016), and two divergent Rickettsiacae species, known as SMLS (*Sitobion miscanthis* L‐type symbiont) and OLO (Orientia‐Like Organism) (Li *et al*., 2011, 2016). Variation in the frequency of aphid endosymbiont infection is thought to arise from a wide range of processes, including aphid utilization of different host plant species, compatibility between different aphid genotypes and symbiont strains, and aphid interactions with the biotic and abiotic environment (Zytynska &Weisser, 2016).

The consequences of endosymbiont infection for aphid fitness are not always clear, particularly for the most recently described taxa. A recent review by Guo *et al*. (2017) summarized the known effects of nine of these endosymbionts, although it is increasingly apparent that these effects are not always consistent between aphid species and endosymbiont strains. A well‐recognized fitness effect of endosymbiotic associations between facultative symbionts and aphid hosts is through their contribution to aphid resistance to parasitoid wasp species, particularly members of the Braconidae, which regulate aphid populations in natural and agricultural vegetation (Oliver *et al*., 2003; Oliver *et al*., 2010; Asplen *et al*., 2014; Cayetano & Vorburger, 2015). The primary mechanism of resistance against Braconid wasps in the pea aphid, *Acyrthosiphon pisum* (Harris), has been attributed to the *Acyrthosiphon pisum Secondary Endosymbiont* (*APSE*) bacteriophage that is frequently associated with *H. defensa* (Moran *et al*., 2005; Degnan & Moran, 2008a,b; Oliver *et al*., 2009). Phage‐derived factors have been reported to arrest the development of wasp embryos (Brandt *et al*., 2017). By contrast, resistance of the peach‐potato aphid, *Myzus persicae* (Sulzer), to Braconid wasps was associated with the facultative endosymbiont *R. insecticola* (von Burg *et al*., 2008; Vorburger *et al*., 2010). Experimental transfer of *R. insecticola* from *M. persicae* confirmed that this strain conferred resistance in *Ac. pisum* to the parasitoid *Aphidius ervi* (Haliday) (although a strain of *R. insecticola* derived from *Ac. pisum* was not protective) and this was attributed to a repertoire of pathogenicity factors in the virulent *R. insecticola* strain (Hansen *et al*., 2012).

The effect of many aphid endosymbionts on their host has been elucidated using *Ac. pisum* as a model. Alongside parasitoid defense, additional traits conferred to aphids by facultative endosymbionts include thermal tolerance (Russell & Moran, 2006) and adaptation to different host plant species (Tsuchida *et al*., 2004); however, in some cases, endosymbiont infection can lead to detrimental effects on aphid fitness as reported for the black bean aphid, *Aphis fabae* (Scopoli) (Vorburger & Gouskov, 2011) and *Ac. pisum* (Martinez *et al*., 2018). Research has also detected differences between aphid species in the effect of some facultative endosymbionts on aphid fitness. For example, the protective effect of *H. defensa* against parasitoid wasps is observed consistently for *Ac. pisum* (Oliver *et al*., 2003) and *A. fabae* (Schmid *et al*., 2012) but not for the English grain aphid, *Sitobion avenae* (Fabricius), or the potato aphid, *Macrosiphum euphorbiae* (Thomas) (Łukasik *et al*., 2013a; Clarke *et al*., 2017), which might be due to infection with nonprotective endosymbiont strains or infection with strains of endosymbionts that are ineffective against particular parasitoid species or genotypes (Vorburger & Rouchet, 2016; Dennis *et al*., 2017).

The bird cherry‐oat aphid, *Rhopalosiphum padi* (Linnaeus), is a worldwide agricultural pest of cereals (Leather *et al*., 1989) and a primary vector of economically damaging plant viruses, including *Barley Yellow Dwarf Virus* (*BYDV*) (Valenzuela & Hoffmann, 2015). Cereal yield losses due to *BYDV* infection can reach 35% (Perry *et al*., 2000) and might rise further as *R. padi* is anticipated to become a more persistent agricultural pest under a changing climate (Finlay & Luck, 2011). Despite the economic importance of *R. padi*, the endosymbionts associated with this aphid species, and their effects on aphid fitness, are not well described. Desneux *et al*. (2018) screened 18 *R. padi* lines and Henry *et al*. (2015) screened 11 lines of *R. padi* for the presence of endosymbionts, but neither study found evidence for the presence of facultative bacterial endosymbionts. By contrast, *S. symbiotica* was detected in populations of *R. padi* along the North Belgian coast by de la Peña *et al*. (2014), and Li *et al*. (2011) detected SMLS in *R. padi* collected from Jiangsu province in China. Functional characterization of these facultative endosymbionts in *R. padi* remains to be reported; although the role of SMLS in cereal aphids is not known, in other aphid species *S. symbiotica* has been reported to enhance aphid resistance against parasitoids, often in synergy with *H. defensa* (Oliver *et al*., 2003; Oliver *et al*., 2006). To address this knowledge gap, species‐specific research is needed to elucidate the role of these aphid endosymbionts, particularly in aphid species of agricultural and economic significance.

The primary strategy for controlling insect pests is via the application of insecticidal chemicals. However, due to their widespread environmental impacts (reviewed by Goulson, 2013), and the emergence of pesticide resistance (Field *et al*., 1988; Bass *et al*., 2014; Foster *et al*., 2014), the continued use of pesticides is considered unsustainable (Geiger *et al*., 2010). Alternative pest management solutions could include augmenting biocontrol using natural enemies (Ramsden *et al*., 2017), and plant‐mediated resistance. Mitchell *et al*. (2016) identified several resistance and tolerance traits that could be employed to increase plant resistance to arthropod pests, including physical barriers, chemical defenses, and reduced plant palatability. Indeed, resistance to cereal aphids has been identified in maize (Betsiashvili *et al*., 2015) and wheat (Girvin *et al*., 2017). A recent review by Jarosova *et al*. (2016) suggested that a strategy for tackling cereal aphid and *BYDV* control might lie in comparison of traits of susceptible modern crops with their wild relatives that display partial inherent resistance as a means to advise molecular breeding programmes. A comparative study of barley, *Hordeum vulgare*, and the wild relative, *H. spontaneum*, highlighted differential gene regulation in response to aphid infestation that might explain their differences in aphid susceptibility (Delp *et al*., 2009), although further work is needed to elucidate fully the underlying mechanism(s). Aphid endosymbionts have been reported to influence aphid fitness and adaptation to host plant species and plants that differ in quality (Tsuchida *et al*., 2004; Gauthier *et al*., 2015; Wagner *et al*., 2015). A better understanding of how endosymbionts modify the effects of plant resistance on aphid success might provide insights for improving the sustainability of insect pest management.

The primary aim of this study was to determine the presence and types of facultative endosymbionts associated with *R. padi* genotypes collected from U.K. populations and to ascertain the effects of any detected endosymbionts on aphid fitness. To achieve this, clonal lines were first established from *R. padi* individuals collected in Eastern Scotland and were characterized for aphid genotype and presence of facultative endosymbionts. Secondly, we tested the hypothesis that facultative endosymbionts influence aphid fitness by (i) examining variation in aphid susceptibility to parasitism by the common parasitoid wasp *Aphidius colemani* (Viereck) (Ronquim *et al*., 2004; McClure & Frank, 2015) and (ii) quantifying aphid performance on a susceptible modern cultivar of barley, *H. vulgare* cv. Concerto, and a barley wild relative, *H. spontaneum 5* (HsP5), previously described as partially aphid‐resistant (Delp *et al*., 2009). We predicted that poor aphid performance relating to aphid genotype and/or endosymbiont status would be exacerbated on a partially resistant plant host.

## Materials and methods

### Plant material

*Hordeum vulgare* cv. Concerto and *H. spontaneum* 5 (HsP5) seeds were surface‐sterilized by rinsing in 2% (v/v) hypochlorite solution followed by three rinses in deionized water (ddH_2_O). Seeds were then kept moist in the dark. *H. vulgare* cv. Concerto seeds were stratified by incubating at room temperature for 48 h whereas HsP5 seeds were incubated at 4 °C for 14 d. Germinated seedlings were planted into a bulrush compost mix (Bulrush, Northern Ireland) under glasshouse conditions (16 : 8 h light and 20 : 15 °C day : night) until the first true leaf emerged (stage 1.2 on the Zadoks *et al*., 1974 decimal key) when they were used in insect assays.

### Insect rearing

Individual apterous *R. padi* adults collected from cereal crops and grasses in Eastern Scotland, UK, in summer 2013 and summer 2016 were used to establish clonal lines. Cultures were reared on 1‐week‐old barley seedlings (*H. vulgare* cv. Optic; growth stage 1.1–1.2 on the Zadoks scale) contained in ventilated cups. These comprised two Perspex cups (50 mm width × 150 mm depth) placed one inside the other; barley seedling roots were placed into a *c*. 10 mm depth of water in the base of the outermost cup, with the stem inserted through a *c*. 5 mm circular hole in the base of the inner cup, and the cup surface was sealed with a mesh‐ventilated lid. A mixed population of the peach‐potato aphid, *M. persicae* (genotypes F and O; determined to be free from facultative endosymbiont infection by diagnostic PCR screening, as described below for *R. padi*), was reared in ventilated Perspex cages on young oilseed rape plants, *Brassica napus* cv. Mascot (growth stage 2.3–2.5 as determined using the Harper & Berkenkamp, 1975 staging key), produced in the growing medium and conditions described above. Plant material was replaced weekly.

Mummies of the Braconid wasp *A. colemani*, supplied by Fargro (West Sussex, UK), were transferred to plastic ventilated boxes supplied with a food source of 50% (v/v) honey, which is deemed suitable for rearing Hymenopteran parasitoids (Perera & Hemachandra, 2014), soaked into a cotton wool ball. A cohort of emerging wasps (5–7 d old) was transferred to *M. persicae*‐infested oilseed rape plants (growth stage 2.3–2.5, determined using the Harper and Berkenkamp key) enclosed in a fine mesh cage. After 12 d, aphid mummies were collected and transferred to a ventilated plastic box supplied with honey solution until the next generation of adult wasps had emerged. To ensure parasitoids had no prior experience of the experimental *R. padi* clones, wasps were reared through at least three generations on *M. persicae* before being used in bioassays. All insect cultures were maintained at 18 ± 2 °C and 16 h : 8 h (day : night).

### Rhopalosiphum padi genotyping

DNA was extracted from frozen homogenized tissue of *c*. 20 aphids per clonal line, using the DNeasy Plant Mini Kit (Qiagen, UK) following the manufacturer's protocol. First, aphids were washed in 96%–100% ethanol (Sigma‐Aldrich, UK) for 5 min and rinsed three times with Gibco® distilled water (ThermoFischer Scientific, UK); samples were then flash‐frozen in liquid nitrogen and homogenized using a micropestle. Extracted DNA was quantified using a Nanodrop ND‐1000 (ThermoFischer Scientific, UK).

Asexual aphid lines were assigned to genotypes based on the length of six of the polymorphic microsatellite markers for *R. padi* identified by Simon *et al*. (2001) and an additional unpublished marker; two other microsatellite markers (R 1–35 and R 3–171; Simon *et al*., 2001) could not be amplified consistently across all asexual lines and were not used. Microsatellite primers are shown in Table S1. A ProFlex PCR System (Applied Biosystems, UK) was used to amplify the target microsatellites in 25 µL reactions, containing final reaction concentrations of 1.5 mmol/L MgCl_2_, 250 µmol/L of mixed deoxynucleotide triphosphate (dNTP), 1 µmol/L forward primer with a 6‐FAM fluorophore attached to the 5′ end, 1 µmol/L reverse primer, 1× Clear GoTaq® reaction buffer (Promega, UK), and 1.25 U GoTaq® DNA Polymerase (Promega, UK), with approximately 15 ng of DNA template. Thermocycling conditions consisted of 98 °C for 30 s, followed by 35 cycles of 98 °C for 30 s, an annealing step consisting of a temperature of either 52 °C or 60 °C for 30 s, and 72 °C for 45 s with a final extension step at 72 °C for 7 min; marker R 6‐3 was annealed at 52 °C, while all other markers were annealed at 60 °C.

Following successful amplification, which was determined by separating a 10 µL aliquot of the amplicons on 2% agarose gel stained with SYBR Safe®, PCR products were separated by capillary electrophoresis; first, the amplified products were diluted 1 : 10 with Gibco® distilled water, then 1 µL of the diluted sample was mixed with 0.16 µL of GeneScan™ 500 ROX™ dye size standard (ThermoFischer Scientific, UK) and suspended in 8.84 µL Hi‐Di™ Formamide (ThermoFischer, UK) in a nonskirted 96‐well plate and sealed with an adhesive film. PCR products were separated on an ABI 3730 DNA Analyser (Applied Biosystems, UK). Product size (bp) was assessed using Peak Scanner™ software v 1.0 (Applied Biosystems, UK), and aphid genotype was determined based on the pattern of PCR product sizes from the amplified alleles (Table S2).

### Facultative endosymbiont detection

#### Diagnostic PCR screening

A diagnostic PCR screen was used targeting universal eubacterial 16S rDNA and the 16–23S rDNA (including the intergenic spacer), and the specific 16S rDNA target sequence of the seven most frequently detected aphid endosymbionts *Regiella insecticola*, *Hamiltonella defensa*, *Serratia symbiotica*, *PAXS*, *Spiroplasma* sp., *Rickettsia* sp., and *Rickettsiella* sp. Initially, extracted aphid DNA was pooled, using 5 µL of DNA from each *R. padi* asexual line, and screened for all diagnostic targets of aphid facultative endosymbionts (see Table S1 for primer details). The reactions were conducted using a G‐storm GS4822 thermocycler in a final reaction volume of 25 µL, with reaction concentrations of 1.5 mmol/L MgCl_2_, 250 µmol/L of mixed dNTP's, 1 µmol/L forward primer, 1 µmol/L reverse primer, 1× Green GoTaq® reaction buffer (Promega, UK) and 1.25 U GoTaq® DNA Polymerase (Promega, UK), and with approximately 15 ng of DNA template; thermocycling conditions are described in Table S3. An aliquot (10 µL) of the amplified product was separated and visualized on 1.5% agarose gel using SYBR Safe® DNA staining agent. In positive reactions, the residual 15 µL of amplified product was purified using the QIAquick PCR Purification Kit (Qiagen, UK) following the manufacturer's protocol. Purified products were quantified and analyzed for quality using a Nanodrop ND‐1000 (ThermoFischer Scientific, UK) and aliquots (250 ng template per 1.5 Kb product length) were prepared for sequencing using Sanger methodology. Sequencing reactions contained 1 µL primer (10 µmol/L), 2 µL of BigDye™ Terminator v3.1 mix (ThermoFisher Scientific, UK), and 1.0 µL of 5× BigDye™ dilution buffer (ThermoFischer Scientific, UK). Cycling was carried out on a Tetrad Cycler (Biorad, Hertfordshire, UK) using the following conditions: 96 °C for 20 s followed by 25 cycles of 96 °C for 10 s, 50 °C for 5 s, 60 °C for 4 min. PCR products were purified by ethanol precipitation, air‐dried, and resuspended in 10 µL of Hi‐Di™ formamide (ThermoFisher Scientific, UK). Sequencing of products was carried out using a 36 cm capillary array on a 48 capillary ABI 3730 (ThermoFisher Scientific, UK).

Sequence data were subjected to a BLASTn search, using the NCBI online database, to check similarity to known aphid endosymbionts. The presence of detected endosymbionts in individual aphid lines was confirmed using diagnostic PCR of the appropriate 16S rDNA gene, and products from positive amplifications were purified and sequenced as described above.

#### 16–23S rDNA sequencing for screening of endosymbionts not targeted by diagnostic PCR

Briefly, the 16–23S rDNA region of a pooled *R. padi* DNA sample was amplified using the thermocycling conditions described above. Amplified products were purified using the QIAquick PCR Purification Kit (Qiagen), following the manufacturer's protocol. To the purified DNA template, a ‐CACC‐ tag was cloned into the 5′ region using the altered 16–23S rDNA F primer: 5′‐CACC AGTTTGATCATGGCTCAGATTG‐3′; this cloning procedure was carried out in a G‐storm GS4822 thermocycler in a final volume of 25 µL containing Phusion® High‐Fidelity DNA Polymerase (0.02 U/µL), 1× High‐Fidelity Buffer (ThermoFisher Scientific, UK), 200 µmol/L of each dNTP, and 0.5 µmol/L of each primer, under the following thermocycling conditions: 98 °C for 3 min, followed by 35 cycles of 98 °C for 30 s, 67 °C for 45 s, and 72 °C for 45 s, with a final elongation step of 72 °C for 10 min. The amplified 5′‐tagged 16–23S rDNA region was purified using the QIAquick PCR Purification Kit (Qiagen) following the manufacturer's instructions. The purified product was cloned into a pENTR™/D‐TOPO® vector with kanamycin resistance and transformed into One Shot® Chemically Competent *E. coli* Cells, following the instructions in the pENTR™ Directional TOPO® Cloning Kit manual (ThermoFisher Scientific, UK).

Transformed *E. coli* were incubated on Luria–Bertani (LB) plates supplemented with 50 µL/mL kanamycin for 16 h at 37 °C; this step was repeated twice to isolate individual colonies. Five individual colonies were selected from each of 25 plates and individually grown in 5 mL of LB broth, supplemented with 50 µL/mL kanamycin, for 16 h at 300 r/min and 37 °C. Plasmids were extracted from 4 mL of the resulting LB culture using the QIAprep Miniprep Kit (Qiagen, UK) following the manufacturer's protocol. Extracted plasmid DNA was quantified and checked for quality using a Nanodrop ND‐1000 (ThermoFischer Scientific, UK), and aliquots (at least 250 ng of template per 5 Kb vector) were subject to Sanger sequencing. The reaction mix comprised 1 µL M13F primer 5′‐GTAAAACGACGGCCAG‐3′ (10 µmol/L), 2 µL of BigDye™ Terminator v3.1 mix (ThermoFisher Scientific, UK) and 1 µL of 5× BigDye™ dilution buffer (ThermoFischer Scientific, UK). The reaction was carried out using a Tetrad Cycler (Biorad, Hertfordshire, UK) with the following conditions: 96 °C for 20 s, followed by 25 cycles of 96 °C for 10 s, 50 °C for 5 s, 60 °C for 4 min. Sequencing products were purified by ethanol precipitation, then air‐dried and resuspended in 10 µL of Hi‐Di™ formamide (ThermoFisher Scientific). Sequencing was carried out using a 360 mm capillary array on a 48 capillary ABI 3730 (ThermoFisher Scientific, UK). Sequences were compared with known prokaryotic sequences held on the NCBI BLAST server.

#### Detection of the APSE bacteriophage

Aphid lines harboring *H. defensa* were subjected to additional diagnostic PCR screening for the detection of the lysogenic bacteriophage, *APSE*, using three *APSE* genomic markers (P3, P35, and P51) using the thermocycling conditions described in Table S3. Amplified PCR products were purified, visualized on 1.5% agarose gel, and sequenced as described above. The best BLASTn hits of the sequences are shown in Table S4.

#### Aphid parasitism assay

Parasitism assays were conducted on four clonal lines of a single aphid genotype (genotype E), two lines harboring *H. defensa* (DL 16/04, DL 16/05) and two lines free from *H. defensa* infection (DL 16/06, DL 16/13), with a total of seven assays per aphid line. Assay arenas were constructed using four leaves of barley (*H. vulgare* cv. Optic, growth stage 1.1–1.2 on the Zadoks scale) fixed adaxial side up into 1% (w/v) agarose in Petri dishes of 120 mm diameter. Ten *R. padi* nymphs (1st–3rd instar) were transferred into each arena and a single *A. colemani* female (5–7 d old), presumed mated, was introduced. After each wasp oviposition event, the attacked nymph was transferred to fresh leaves of *H. vulgare* cv. Optic in a ventilated cup. Attacked nymphs were examined daily for 12 d postparasitism. Mummies were carefully removed using fine forceps and placed in ventilated plastic boxes until eclosion. *Rhopalosiphum padi* mortality was measured as the proportion of nymphs mummified out of the ten nymphs that had been attacked, and sex determination of the emerged wasps was scored based on the presence of an ovipositor.

#### Aphid performance assays

Three separate aphid performance experiments were carried out, using two experimental methods, on plants initially at the first true leaf stage (1.2 on the Zadoks decimal growth scale; Zadoks *et al*., 1974), which were conducted as follows.

Experiments one and two each consisted of 12 replicates for each experimental treatment, and used Perspex clip cages (MacGillivray & Anderson, 1957) to contain the aphids onto the experimental plants; these two experiments were conducted under glasshouse conditions (16 h : 8 h L : D and 20 : 14 °C) and assessed the performance of the same four genotype E aphid lines with differential *H. defensa* infection that were used in the parasitism assays (see above and Table 1). Plants were infested with a single apterous aphid, which was allowed to reproduce overnight. A total of three nymphs were retained on each plant and mean nymph mass was recorded after 48 h and 144 h, after which a single nymph, selected at random, was returned to the plant; for this focal nymph, data was collected on the prereproductive period (*d*) and the intrinsic rate of population increase (*r_m_*).

**Table 1:** Presence of *Hamiltonella defensa* and *APSE* in 16 asexual lines of *R. padi*

		Endosymbiont marker				*APSE* marker		
*R. padi* asexual line	Genotype	16S rDNA (+ve for *B. aphidicola*)	16–23S rDNA (+ve for facultative symbiont presence)	*H. defensa* 16S rDNA	Accession number of sequenced *H. defensa* 16S rDNA	*APSE* P51	*APSE* P35	*APSE* P3
AK 13/33 ‡	A	**+**	–	–				
AK 13/34 ‡	B	**+**	–	–				
DL 16/14	C	**+**	**+**	**+**	MG595523	–	**+**	–
DL 16/12	D	**+**	–	–				
DL 16/02	E	**+**	–	–				
DL 16/03	E	**+**	**+**	**+**	MG595518	**+**	**+**	**+**
DL 16/04 †, ‡	E	**+**	**+**	**+**	MG595519	**+**	**+**	**+**
DL 16/05 †, ‡	E	**+**	**+**	**+**	MG595520	**+**	**+**	**+**
DL 16/06 †, ‡	E	**+**	–	–				
DL 16/07	E	**+**	**+**	**+**	MG595521	**+**	**+**	**+**
DL 16/08	E	**+**	**+**	**+**	MG595522	**+**	**+**	**+**
DL 16/10	E	**+**	–	–				
DL 16/13 †, ‡	E	**+**	–	–				
DL 16/15	F	**+**	–	–				
DL 16/16	F	**+**	–	–				
JB	G	**+**	**−**	**−**				

^†^Indicates aphid lines used in parasitism experiments.

^‡^Indicates aphid lines used in performance experiments.

Experiment three assessed the performance of aphid lines representing aphid genotypes A, B, and E, consisted of 10 replicates per experimental treatment and was conducted in a Sanyo controlled environment cabinet (PAR 150 µmol/m^−2^ s^−1^, 16 : 8 h L : D and 20 °C ± 2 °C); the same four genotype E aphid lines used in experiments one and two were used, along with genotype A (aphid line AK 13/33) and genotype B (AK 13/34), and aphids were contained on plants using microperforated bags. Plants were infested with a single apterous aphid, which was allowed to reproduce overnight. The entire progeny of the aphid was retained on the plant for 48 h, at which point all nymphs were removed and the mass of a single nymph, selected at random, was recorded and returned to the plant for further monitoring of nymph mass at 144 h, prereproductive period and *r_m_*. Aphid survival was also recorded in experiment 3 until aphids were 21 d old.

For all three experiments, nymph mass gain was calculated as the change in mass between 144 and 48 h. Aphid *r_m_* was calculated using the equation of Wyatt and White (1977), where *d* is the time period between aphid birth and production of first progeny, and *Fd* is the total progeny over a time period equal to *d*:

rm=0.74 ln Fdd.

#### Statistical analysis

All statistical analyses were carried out using R Studio Desktop version 1.0.143 running R version 3.4.0 (R Core Team, 2014), with additional packages broom (v. 0.4.2) (Robinson, 2017), car (v. 2.1–4) (Fox & Weisberg, 2011), coxme (v. 2.2–7) (Therneau, 2018), ggplot2 (v. 2.2.1) (Wickham, 2009), ggpubr (v. 0.1.2) (Kassambara, 2017), lme4 (v. 1.1–13) (Bates *et al*., 2015), lmerTest (v. 2.0–33), pkbrtest (v.0.4–7) (Halekoh & Højsgaard, 2014), pastecs (v. 1.3–18) (Grosjean & Ibanez, 2014), survival (v. 2.41–3) (Therneau & Grambsch, 2000), and survminer (v. 0.3.1) (Kassambara & Kosinski, 2017).

Aphid mortality (mummification) and the proportion of female : male wasps emerging from the mummified aphids were each modeled using a generalized linear mixed effects model fitted with binomial distribution with wasp generation and batch incorporated as random factors and aphid asexual line as a random factor nested within endosymbiont association. Model simplification in both cases was carried out through manual backward stepwise model selection. Throughout both model simplification processes, analysis of deviance using a Type II Wald *χ* ^2^ test and observing changes in AIC ensured that model simplification was justified and that the data fitted the model parameters. Fitted‐residual plots of the final models were assessed for model suitability.

Aphid performance data were split into two subDatasets. One, labeled “Genotype,” assessed insect performance in relation to aphid genotype, host plant (cv. Concerto or HsP5) and genotype × host plant interaction in aphid lines uninfected with *H. defensa*, namely AK 13/33, AK 13/34, DL 16/06, and DL 16/13 belonging to genotypes A, B, E, and E, respectively. Another subDataset, labeled “Endosymbiont,” assessed aphid performance in relation to host plant, facultative endosymbiont association with *H. defensa* and the plant × endosymbiont interaction in four aphid lines from genotype E with differential *H. defensa* infection: DL 16/04 (*Hd* +), DL 16/05 (*Hd* +), DL 16/06 (*Hd*–), and DL 16/13 (*Hd*–). In each subDataset, nymph mass gain, prereproductive period and *r_m_* were assessed in separate linear mixed effects models, incorporating experiment number and experimental block as random factors. To minimize the influence of multiple aphid lines representing genotype E on the model outcome, aphid line was incorporated into the models as a nested random factor and was nested within aphid genotype in the “Genotype” subDataset and within Endosymbiont association in the “Endosymbiont” subDataset. All models were simplified using manual backward stepwise model selection to reach the final models with Type II Wald *χ* ^2^ analysis of deviance and observing changes in AIC to ensure that model simplification was justified and that the data fitted the model parameters. Calculation of the differences of Least Squares Means was used as a *post hoc* test on the final models to identify which levels in each factor were significantly different.

For survival analysis, the two subDatasets were modeled separately by fitting a Cox proportional hazards regression model with experimental block incorporated as a random factor and aphid line incorporated as a nested factor within aphid genotype or endosymbiont association. Model simplification was carried out using manual backward stepwise model selection.

## Results

### Hamiltonella defensa associates with Rhopalosiphum padi

Based on the banding patterns of the six microsatellite markers used, the *R. padi* asexual lines were grouped into one of seven genotypes (labeled A–G; Table S2). *Hamiltonella defensa* was detected in six asexual lines within genotypes C and E only (Table 1). These six aphid lines were also positive for the *APSE* P35 genomic marker, with five lines positive for all three *APSE* genomic markers (P3, P35, P51; Table 1). Additional sequencing of pooled 16–23S rDNA extracted from all aphid lines did not detect the presence of additional eubacterial endosymbionts. Differential presence of *H. defensa* and *APSE* was detected in genotype E only (Table 1); comparative assays to detect the effect(s) of *H. defensa* infection on aphid performance focused on four aphid lines from genotype E, two with and two without *H. defensa* infection (DL 16/04, DL 16/05, DL 16/06, and DL 16/13). Insect fitness was compared between multiple aphid genotypes using *H. defensa*‐free clonal lines of genotypes A (AK 13/33), B (AK 13/34), and E (DL 16/06, DL 16/13).

### Hamiltonella defensa confers protection against A. colemani in R. padi

**Figure 1:**
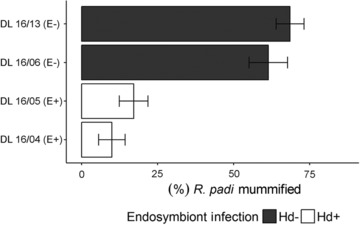
The effect of facultative endosymbiont presence (+/–) in aphid lines of genotype E on aphid mortality after attack by *A. colemani*. Values are means ± SE. Number of observations in model = 28.

Aphid mortality after parasitoid attack was significantly lower for aphid lines harboring the facultative endosymbiont *H. defensa* (*χ* ^2^_1,24_ = 92.07, *P* < 0.001; Fig. 1; Table 2). The ratio of female : male wasps emerging from mummified aphids can be used as an indicator of aphid host suitability, with a higher proportion of female progeny indicative of a good quality host (King, 1987; Pandey & Singh, 1999); the observed female : male ratio was consistent across all treatments (50 : 50, on average) indicating that aphid genotype and presence of *H. defensa* did not alter aphid quality as a host for *A. colemani* (Table 2).

**Table 2:** Summary of statistical modeling outputs for aphid parasitism assays. For each performance parameter, the treatment factor, model basis, error distribution, analysis method, and statistical outputs are shown. Type II Wald *χ* ^2^ analysis of deviance and observation of fitted‐residual plots was conducted throughout the modeling simplification process to ensure data fitted the model parameters and that model simplification was justified

Response variable	Treatmentfactor	Model basis	Error distribution	Modelanalysis	Test statistic	Degrees of freedom (residuals)	*P* value
Susceptibility to parasitism (number of aphid mummies)	Endosymbiont infection	Generalized linear mixed effects model	Binomial	Type II Wald *χ* ^2^ analysis of deviance (*χ* ^2^ Test)	*χ* ^2^ = 92.07	1 (24)	<0.001
Sex of wasp progeny	Endosymbiont infection	Generalized linear mixed effects model	Binomial		*χ* ^2^ = 0.07	1 (100)	0.787

### Aphid genotype and host plant identity are dominant factors influencing aphid performance

**Figure 2:**
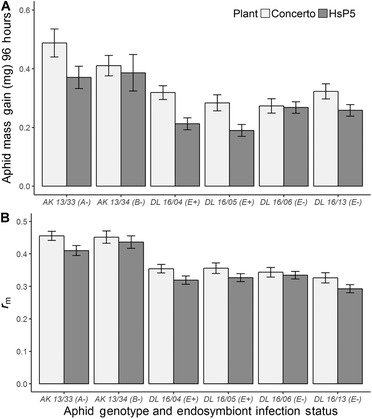
Effects of plant identity, aphid genotype, and *H. defensa* infection (+/**−**) on (A) nymph mass gain (mg) over a 96‐h period and (B) aphid *r_m_*. Values are means ± SE. Number of observations in model = 280. [Correction added on 21 February 2019, after first online publication: Figure 2's image has been replaced.]

Aphid prereproductive period and survival probability were unaffected by host plant identity, aphid genotype, and endosymbiont infection (Tables 3 and 4). However, assessment of the “Genotype” subDataset indicated that host plant identity significantly affected nymph mass gain (*F*_1,130_ = 6.49_,_ *P* = 0.012; Fig. 2A; Table 3) and aphid *r_m_* (*F*_1,104_ = 11.94, *P <* 0.001; Fig. 2B; Table 3), with lowest values on HsP5 compared with cv. Concerto. This effect was also detected in the “Endosymbiont” subDataset (*F*_1,170_ = 31.77, *P* < 0.001; Fig. 2A; Table 4). In addition, nymph mass gain varied significantly between genotypes (*F*_2,131_ = 4.48_,_ *P* = 0.013; Fig. 2A; Table 3) with significantly lower values in genotype E compared with genotype A (Table 5).

**Table 3:** Summary of statistical modeling outputs for aphid performance parameters using the Genotype subDataset. For each performance parameter, the treatment factor, model basis, error distribution or statistical method applied, analysis method, and the statistical outputs are shown. Type II Wald *χ* ^2^ analysis of deviance and observation of fitted‐residual plots was conducted throughout the modeling simplification process to ensure data fitted the model parameters and that model simplification was justified

Response variable	Treatment factor	Model basis	Error distributionor statisticalmethod	Model analysis	Test statistic	Degrees of freedom (Residuals)	*P* value
Nymph mass gain	Genotype	Linear mixed effects model	Maximum Likelihood (ML)	Type III analysis of variance with satterthwaite approximation for degrees of freedom (Type III ANOVA)	*F* = 4.48	2 (131)	0.013
	Plant				*F* = 6.49	1 (130)	0.012
	Genotype × Plant				*F* = 1.01	2 (131)	0.366
Prereproductive Period	Genotype	Linear mixed effects model	ML	Type III ANOVA	*F* = 0.24	2 (109)	0.784
	Plant				*F* = 1.61	1 (94)	0.208
	Genotype × Plant				*F* = 0.15	1 (150)	0.861
*r_m_*	Genotype	Linear mixed effects model	ML	Type III ANOVA	*F* = 0.92	2 (104)	0.454
	Plant				*F* = 11.94	1 (104)	< 0.001
	Genotype × Plant				*F* = 0.78	2 (103)	0.462
Aphid survival	Genotype	Cox proportional hazards regression	N/A	Type II Wald *χ* ^2^ analysis of deviance (*χ* ^2^ Test)	*χ* ^2^ = 0.86	2	0.649
	Plant				*χ* ^2^ = 0.92	1	0.919

**Table 4:** Summary of statistical modeling outputs for aphid performance experiments using the Endosymbiont subDataset. For each performance parameter, the treatment factor, model basis, error distribution or statistical method applied, analysis method, and the statistical outputs are shown. Type II Wald *χ* ^2^ analysis of deviance and observation of fitted‐residual plots was carried out throughout the modeling simplification process to ensure data fitted the model parameters and that model simplification was justified

Response variable	Treatment factor	Model basis	Error distributionor statisticalmethod	Model analysis	Test statistic	Degrees of freedom (residuals)	*P* value
Nymph mass gain	Endosymbiont	Linear mixed effects model	Maximum Likelihood (ML)	Type III analysis of variance with Satterthwaite approximation for degrees of freedom (Type III ANOVA)	*F* = 4.69	1 (203)	0.031
	Plant				*F* = 24.76	1 (203)	< 0.001
	Endosymbiont × Plant				*F* = 5.03	1 (203)	0.026
Prereproductive period	Endosymbiont	Linear mixed effects model	ML	Type III ANOVA	*F* = 2.61	1 (201)	0.464
	Plant				*F* = 2.67	1 (202)	0.104
	Endosymbiont × Plant				*F* = 0.46	1 (202)	0.497
*r_m_*	Endosymbiont	Linear mixed effects model	ML	Type III ANOVA	*F* = 3.10	1 (170)	0.807
	Plant				*F* = 31.77	1 (170)	< 0.001
	Endosymbiont × Plant				*F* = 1.08	1 (170)	0.300
Aphid survival	Endosymbiont	Cox proportional hazards regression	N/A	Type II Wald *χ* ^2^ analysis of deviance (*χ* ^2^ Test)	*χ* ^2^ = 0.14	1	0.713
	Plant				*χ* ^2^ = 2.19	1	0.139

**Table 5:** *Post hoc* test of the least squares means for observed differences in aphid nymph mass gain between aphid genotypes, showing pairwise comparisons (*t* and *P* values) for each set of aphid genotypes

Aphid genotype		Aphid genotype	Fitness parameter	subDataset	*t* value	*P* value
A	vs.	B	Nymph mass gain	Genotype	0.88	0.380
A	vs.	E			2.84	0.005
B	vs.	E			1.81	0.072

### Interactive effects of plant identity and H. defensa presence on R. padi nymph mass gain

Analysis of the “Endosymbiont” subDataset highlighted an endosymbiont × host plant interaction for nymph mass gain, which was due to significantly lower mass gain in *H. defensa*‐infected genotype E nymphs feeding on HsP5 (*F*_1,203_ = 5.03, *P* = 0.026; Fig. 2A; Table 4)

## Discussion

**Figure 3:**
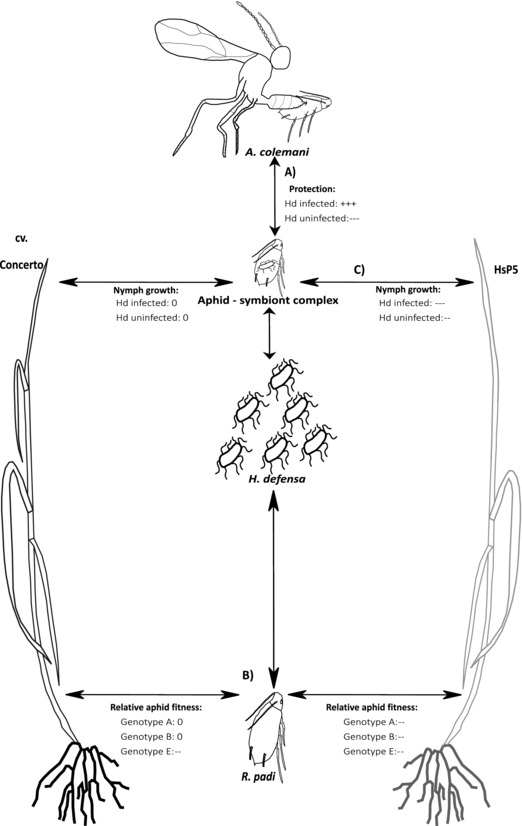
Summary of interactions between aphid genotype, *H. defensa* and other trophic groups. Arrows indicate trophic interactions that had positive (+), neutral (0), or negative (**−**) effects on aphid fitness; the relative magnitude of effect is shown by the number of symbols. (A) *H. defensa* conferred protection against the parasitoid wasp, *A. colemani*. (B) Aphid genotype was the main determinant of aphid fitness on *H. vulgare* cv. Concerto compared with *H. spontaneum* (HsP5). (C) Association with *H. defensa* was detrimental to juvenile aphid growth when feeding on unfavorable host plants.

This study reports on the presence of the facultative bacterial endosymbiont, *Hamiltonella defensa*, in the bird cherry‐oat aphid, *R. padi*, and assesses the effect of this endosymbiont on aphid fitness. Novel data are presented on the association between *R. padi* genotypes and *H. defensa* sampled from U.K. populations. Intraspecific variation in *R. padi* performance was detected in relation to aphid genotype and *H. defensa* infection and led to differential outcomes for aphid interactions with two host plant species and a natural enemy, which are summarized in Figure 3.

### The outcome of a symbiont–aphid–parasitoid relationship is species‐specific

This study provides evidence that *R. padi* forms associations with the facultative endosymbiont *H. defensa*, which was detected in *c*. 38% of the aphid lines assessed and in two out of seven aphid genotypes, although not in all representative lines of these genotypes. Polymorphic associations with *H. defensa* were only detected in genotype E. Previous studies of *R. padi* have not detected *H. defensa* but have reported the presence of two other facultative endosymbionts, *S. symbiotica* (de la Peña *et al*., 2014), and SMLS (Li *et al*., 2011), although their effects on aphid fitness remain to be established. The number of aphid lines infected with *H. defensa* in this study (6 out of a total of 16 lines) is representative of the intermediate infection frequencies detected for heritable symbionts in aphid populations (Russell *et al*., 2013).

To date, the most frequent effect on aphid fitness attributed to *H. defensa* is resistance to Hymenopterous parasitoids, primarily Braconid wasps (Oliver *et al*., 2010) such as *A. ervi* attacking *Ac. pisum* (Oliver *et al*., 2003) and *Lysiphlebus fabarum* (Marshall) attacking *Ap. fabae* (Schmid *et al*., 2012). Consistent with these studies, our findings show that the *H. defensa–APSE* complex can provide protection to *R. padi* against the parasitoid wasp *A. colemani* (Fig. 3A) and reinforces the defensive role attributed to this symbiont.

However, endosymbiont‐conferred protection is not necessarily observed consistently against all potential parasitoids of an aphid species. McLean and Godfray (2015) assessed the efficacy of endosymbiont‐mediated resistance to Braconid and Aphelinid wasps in relation to *H. defensa* strains selected from *Ac. pisum* biotypes adapted to different host plants. The authors detected differences in parasitism susceptibility due to *H. defensa* strain, with one strain able to confer protection against the Aphelinid wasp *Aphelinus abdominalis* (Dalman), but unable to provide resistance to the Braconid wasp *A. ervi*. Differences in parasitoid wasp susceptibility were also attributed to aphid biotype, indicating that aphid adaptation to host plant species could influence the efficacy of endosymbiont‐mediated resistance. A hypothesis was put forward by Hopper *et al*. (2018) to explain why *H. defensa* does not confer widespread protection against Aphelinid wasps, and relates to the anhydropic chlorinated eggs produced by the Aphelinidae, which are thought to be less susceptible to secreted *APSE* toxins.

Additionally, the cowpea aphid, *Aphis craccivora* (Koch), is attacked by a number of Braconid wasps, including *Binodoxys communis* (Gahan), *B. koreanus* (Stary), *L. orientalis* (Stary & Rakhshani), and *A. colemani*; *H. defensa* infection did not protect aphids against *L. orientalis* or *A. colemani*, but did provide resistance to *B. communis* and *B. koreanus* (Asplen *et al*., 2014). The authors hypothesized that differential protection conferred by endosymbionts against particular parasitoid species might be linked to particular *H. defensa*–*APSE* combinations, and recent work provides evidence for specificity of symbiont defense in relation to *APSE* strain and aphid and parasitoid genotype (Dennis *et al*., 2017; Käch *et al*., 2018; Martinez *et al*., 2018). These factors could explain why *H. defensa* provided protection to *R. padi* against *A. colemani* (this study) and to *Ap. fabae* against *A. colemani* (Cayetano & Vorburger, 2015), but did not protect *Ap. craccivora* against *A. colemani* (Asplen *et al*., 2014).

The *APSE* genome has been reported to undergo rapid recombination resulting in strain‐dependent variation in the identity of toxins and their protective effects (Degnan & Moran, 2008a,b; Dennis *et al*., 2017), which might also explain differences between wasp genera in their susceptibility to symbiont–APSE‐mediated protection (Käch *et al*., 2018). Other studies have highlighted the existence of aphid‐encoded resistance to parasitism irrespective of endosymbiont presence in *M. euphorbiae* and *Ac. pisum* (Martinez *et al*., 2014; Clarke *et al*., 2017), indicating that aphid‐encoded traits could be another factor influencing the specificity of parasitoid resistance in aphids. Experimental manipulation of symbiont infection in different aphid genotypes would be a useful next step to identify the contribution of these factors to the defensive phenotype in *R. padi*.

### Aphid genotype is a key determinant of aphid fitness

Intraspecific variation in *R. padi* mass gain in the present study was attributed mainly to aphid genotype and not to *H. defensa* infection (Fig. 3B), with individuals belonging to genotype E generally performing poorly compared with genotypes A and B. Aphid genotype is often identified as a key determinant of aphid performance, for example in *S. avenae* (Figueroa *et al*., 2004) and *M. euphorbiae* (Karley *et al*., 2017). A recent study reported that *M. euphorbiae* genotypes capable of forming endosymbiotic associations with *H. defensa* had higher fitness than those genotypes which did not support *H. defensa* infection, at least when feeding on a susceptible host plant species (Clarke *et al*., 2017), which contrasts with the findings for *R. padi* lines assessed in the current study. Differential effects of aphid genotype on fitness might also depend, however, on plant suitability for aphids (Karley *et al*., 2017). In general, all *R. padi* genotypes examined in the present study performed poorly on the wild species HsP5 compared with the commercial barley cultivar Concerto, indicating that HsP5 is partially resistant to aphids irrespective of aphid genetic variation. To understand the implications of these findings in the context of pest control, further work is needed to assess whether the frequency of *R. padi* genotypes detected in this study are representative of field populations.

### Endosymbiont infection exacerbates the effects of poor plant quality

Infection with *H. defensa* has previously been shown to decrease the longevity of *Ap. fabae* (Vorburger & Gouskov, 2011). Although we did not identify any negative effects of *H. defensa* infection on aphid longevity, a key finding of this study was that symbiont‐infected individuals exhibited reduced growth during their juvenile stages compared with symbiont‐free individuals, but only on the partially resistant plant, HsP5 (Fig. 3C), in line with our original prediction. The mechanism of aphid resistance in HsP5 has not been fully characterized, but partial resistance to aphids in the wild relative of wheat, *Triticum monococccum*, is thought to be phloem‐mediated, linked to increased secondary metabolite concentrations (Greenslade *et al*., 2016). Whatever the causal mechanism of resistance, it is possible that the decrease in nymph growth rate on HsP5 in aphid lines harboring *H. defensa* resulted from resource demand by the endosymbiont, which intensified the negative effects of feeding on a poor quality plant host. Indeed a similar observation was made by Chandler *et al*. (2008), where growth of *Ap. fabae* differed between two host plants—a favorable host (*Vicia faba*) and an unfavorable host (*Lamium purpureum*). The negative effect of *L. purpureum* on aphid fitness was exacerbated by the presence of the facultative endosymbionts *Regiella insecticola* and *H. defensa*. This observation was thought to relate to low phloem concentrations of amino acids in *L. purpureum*, which disrupted the ability of the aphid to regulate facultative endosymbiont titres in aphid tissues, leading to greater symbiont resource demand and decreased insect growth (Chandler *et al*., 2008). The possibility that the symbiont‐associated decrease in nymph growth of *R. padi* on HsP5 is linked to symbiont resource demand and poor quality phloem sap is an interesting avenue for further research, especially if it reveals a trade‐off with aphid resistance to parasitoid wasps.

## Conclusions

This study highlights both large and small magnitude effects of a facultative endosymbiont on aphid fitness that could influence aphid ecology and population dynamics by modifying the outcome of aphid interactions with host plants and natural enemies. Our findings show that infection with the defensive endosymbiont *H. defensa* provides protection to *R. padi* against a common parasitoid wasp through a 5 fold increase in aphid survival after parasitoid attack (Fig. 3A). However, this benefit could be partly mitigated by the 16% reduction, on average, in growth of symbiont‐infected nymphs observed on a partially resistant host plant (Fig. 3C), although this might be a relatively small price to pay for parasitoid protection. Finally, while most genotypes exhibited reduced fitness on the partially resistant host (Fig. 3B), the fittest genotypes still performed better on this host than the least fit genotypes. In summary, these findings suggest that plant resistance factors will favor the fittest *R. padi* genotypes, but symbiont‐infected individuals will be favored when parasitoids are abundant (Käch *et al*., 2018), although these aphids might not achieve optimal performance on a poor quality host plant. While the consequences of symbiont‐conferred parasitoid resistance for aphid biocontrol are increasingly recognized (Vorburger, 2018), symbiont‐mediated fitness trade‐offs that interact with plant defensive traits have received relatively little attention until recently (e.g., Frago *et al*., 2017; Karley *et al*., 2017) and should be taken into account when deploying crop resistance and natural enemies for integrated management of crop pests.

## Disclosure

The authors declare no conflicts of interest.

## Data archiving and deposition

16S rDNA sequences of the sequenced *H. defensa* strains from the *R. padi* lines assessed have been deposited into GenBank within the National Centre for Biotechnology Information database, with accession numbers MG595518–MG595523.

## Supporting information


**Table S1** Primer names, targets, 5′–3′ sequence, use, and source for all primers used in this study for genotyping *R. padi* asexual lines using microsatellite markers and for facultative endosymbiont screens.
**Table S2** *R. padi* genotyping results showing aphid asexual line, collection site, original collection host plant, assigned genotype, and the allele sizes (in bp) for seven microsatellite loci.
**Table S3** Thermocycling conditions for diagnostic PCR of aphid facultative endosymbionts and *APSE* amplification.
**Table S4** Best BLASTn hits for *APSE* endosymbiont marker sequences amplified from *R. padi* lines infected with *H. defensa*. Sequences were analyzed for BLASTn similarity against sequences held on the NCBI database.
