# Supplementary material for: The price of protection: a defensive endosymbiont impairs nymph growth in the bird cherry‐oat aphid, Rhopalosiphum padi
Source: Insect Sci. 2018 Jul 25;27(1):69–85. doi: 10.1111/1744-7917.12606 (PMC7379937; doi:10.1111/1744-7917.12606)
Supplement: Supplementary file 1 — Table S1 Primer names, targets, 5′–3′ sequence, use, and source for all primers used in this study for genotyping R. padi asexual lines using microsatellite markers and for facultative endosymbiont screens. Table S2 R. padi genotyping results showing aphid asexual line, collection site, original collection host plant, assigned genotype, and the allele sizes (in bp) for seven microsatellite loci. Table S3 Thermocycling conditions for diagnostic PCR of aphid facultative endosymbionts and APSE amplification. Table S4 Best BLASTn hits for APSE endosymbiont marker sequences amplified from R. padi lines infected with H. defensa. Sequences were analyzed for BLASTn similarity against sequences held on the NCBI database. [file INS-27-69-s001.docx]

**Table S1** Primer names, targets, 5’–3’ sequence, use and source for all primers used in this study for genotyping *R. padi* asexual lines using microsatellite markers and for facultative endosymbiont screens.

| **Primer Name** | **Target** | **Sequence (5'–3')** | **Use in This Study** | **Primer Source** |
| --- | --- | --- | --- | --- |
| R5.10_F | R5.10 Microsatellite Marker (Accession: AF277462.1) | [6FAM]CCGACTAAGCTTAATATTGTTTG | *R. padi* Genotyping | Simon et al. (2001) |
| R5.10_R |  | CGGTTCGGAGAACATAAGAG |  |  |
| R2.73_F | R2.73 Microsatellite Marker (Accession: AF277466.1) | [6FAM]CGTAGACCGCCGCGGG | *R. padi* Genotyping |  |
| R2.73_R |  | GTCGTTTCTGGTCAGCGGCC |  |  |
| R5.29.b_F | R5.29.b Microsatellite Marker (Accession: AF277464.1) | [6FAM]CATGAGTGTGTCCCTTTTAAC | *R. padi* Genotyping |  |
| R5.29.b_R |  | GATGGACGAGGGGACAC |  |  |
| R6.3_F | R6.3 Microsatellite Marker (Accession: AF277465.1) | [6FAM]CGAAATGTACCCACTATAAAC | *R. padi* Genotyping |  |
| R6.3_R |  | CAAATTTAAATGTATAATCAATG |  |  |
| R5.138_F | R5.138 Microsatellite Marker (Accession: AF2909080.1) | [6FAM]TATACACGCTCGCGCTTACG | *R. padi* Genotyping |  |
| R5.138_R |  | CCGAGCACGAATTGTTCC |  |  |
| R5.50_F | R5.50 Microsatellite Marker (Accession: AF290979.1) | [6FAM]TGTTACGCGGAGTGTGTAGG | *R. padi* Genotyping |  |
| R5.50_R |  | CCACAGAGCGTTGTCATC |  |  |
| R4.140_F | R4.140 Microsatellite Marker (Accession: AF277460.1) | [6FAM]CAGTATAAATGGTGATAACGGAAAA | *R. padi* Genotyping | Designed for This Study |
| R4.140_R |  | CGGTCGACTATATATTTGGTACT |  |  |
| 16SA1 | 16S rDNA (Positive for bacterial presence/aphid primary endosymbiont) | AGAGGTTGATCMTGGCTCAG | Facultative Endosymbiont Screen | Fukatsu & Nikoh (2000) |
| 16SB1 |  | TACGGYTACCTTGTTACGACTT |  |  |
| 10F | 16-23S rDNA Including Intergenic Spacer Region (positive for secondary endosymbionts) | AGTTTGATCATGGCTCAGATTG | Facultative Endosymbiont Screen | Sandström et al., 2001 |
| 480R |  | CACGGTACTGGTTCACTATCGGTC |  |  |
| 16SA1 | *Regiella insecticola* 16S rDNA | AGAGGTTGATCMTGGCTCAG | Facultative Endosymbiont Screen | Fukatsu & Nikoh (2000) |
| PASScmp |  | GCAATGTCTTATTAACACAT |  | Fukatsu et al (2000) |
| PABSF | *Hamiltonella defensa* 16S rDNA | AGCGCAGTTTACTGAGTTCA | Facultative Endosymbiont Screen | Darby & Douglas (2003) |
| 16SB1 |  | TACGGYTACCTTGTTACGACTT |  | Fukatsu & Nikoh (2000) |
| U99F | *Serratia symbiotica* 16S rDNA | ATCGGGGAGTAGCTTGCTAC | Facultative Endosymbiont Screen | Sandström et al., 2001 |
| 16SB1 |  | TACGGYTACCTTGTTACGACTT |  | Fukatsu & Nikoh (2000) |
| PAXSF | *PAXS* 16S rDNA | GAAGCAATGCAAAGAGTGTTGC | Facultative Endosymbiont Screen | Guay et al (2009) |
| 1507R |  | TACCTTGTTACGACTTCACCCCAG |  | Sandström et al., 2001 |
| 16SA1 | *Rickettsia sp.* 16S rDNA | AGAGGTTGATCMTGGCTCAG | Facultative Endosymbiont Screen | Fukatsu & Nikoh (2000) |
| Rick16SR |  | CATCCATCAGCGATAAATCTTTC |  | Fukatsu et al (2000) |
| 16SA1 | *Spiroplasma sp.* 16S rDNA | AGAGGTTGATCMTGGCTCAG | Facultative Endosymbiont Screen | Fukatsu & Nikoh (2000) |
| TKSSsp |  | TAGCCGTGGCTTTCTGGTAA |  |  |
| RCL16-211F | *Rickettsiella sp.* 16S rDNA | GGGCCTTGCGCTCTAGGT | Facultative Endosymbiont Screen | Tsuchida et al (2010) |
| RCL16S-470R |  | TGGGTACCGTCACAGTAATCGA |  |  |
| APSE20.8F | *APSE* P35 Gene | GCCGCGGGGCGTGTTATTGACG | *APSE* Screen | Degnan & Moran (2008) |
| APSE21.7R |  | TTAAGGCCCGCTCATAAGCTG |  |  |
| APSE34.0F | *APSE* P51 Gene | AGGTGCGATTACCCTGTTTG | APSE Screen |  |
| APSE34.9R |  | GATAAAACATCGCCGTTTGC |  |  |
| APSE1.1F | *APSE* P3 Gene | TCGGGCGTAGTGTTAATGAC | APSE Screen |  |
| APSE2.4R |  | TTCCATAGCGGAATCAAGG |  |  |
| 10F_Seq | 16-23S rDNA Including Intergenic Spacer Region | CACCAGTTTGATCATGGCTCAGATTG | 16-23S rDNA Sequencing | Designed for This Study |
| 480R |  | CACGGTACTGGTTCACTATCGGTC |  | Fukatsu & Nikoh (2000) |

**Table S2**: *R. padi* genotyping results showing aphid asexual line, collection site, original collection host plant, assigned genotype and the allele sizes (in bp) for seven microsatellite loci.

| ***R. padi* Asexual Line** | **Collected From** | **Host Plant** | **Genotype Assigned** | **Microsatellite Marker allele sizes (bp)** | | | | | | | | | | | | | |
| --- | --- | --- | --- | --- | --- | --- | --- | --- | --- | --- | --- | --- | --- | --- | --- | --- | --- |
|  |  |  |  | **R 5-10** | | **R 2-73** | | **R 5-29.b** | | **R 6-3** | | **R 5-138** | | **R 5-50** | | **R 4-140** | |
| AK 13/33 | Balruddery | *H. vulgare* | A | 253 | 263 | 246 | 262 | 184 | 192 | 176 | | 246 | 264 | 311 | 315 | 142 | 146 |
| AK 13/34 | Balruddery | *H. vulgare* | B | 259 | 261 | 246 | 272 | 163 | 191 | 176 | | 266 | 276 | 345 | 365 | 142 | 146 |
| DL 16/14 | Invergowrie | *H. vulgare* | C | 258 | 262 | 246 | 262 | 163 | 191 | 176 | | 266 | 276 | 345 | 365 | 142 | 146 |
| DL 16/12 | Invergowrie | *H. vulgare* | D | 258 | 262 | 246 | 256 | 163 | 191 | 175 | 177 | 266 | 276 | 345 | 365 | 142 | 146 |
| DL 16/02 | Invergowrie | *Poa annua* | E | 258 | 262 | 246 | 262 | 170 | 174 | 175 | 177 | 240 | 248 | 323 | 339 | 142 | 146 |
| DL 16/03 | Invergowrie | *P. annua* | E | 258 | 262 | 246 | 262 | 170 | 174 | 175 | 177 | 240 | 248 | 323 | 339 | 142 | 146 |
| DL 16/04 | Invergowrie | *P. annua* | E | 258 | 262 | 246 | 262 | 170 | 174 | 175 | 177 | 240 | 248 | 323 | 339 | 142 | 146 |
| DL 16/05 | Invergowrie | *P. annua* | E | 258 | 262 | 246 | 262 | 170 | 174 | 175 | 177 | 240 | 248 | 323 | 339 | 142 | 146 |
| DL 16/06 | Invergowrie | *P. annua* | E | 258 | 262 | 246 | 262 | 170 | 174 | 175 | 177 | 240 | 248 | 323 | 339 | 142 | 146 |
| DL 16/07 | Invergowrie | *P. annua* | E | 258 | 262 | 246 | 262 | 170 | 174 | 175 | 177 | 240 | 248 | 323 | 339 | 142 | 146 |
| DL 16/08 | Invergowrie | *P. annua* | E | 258 | 262 | 246 | 262 | 170 | 174 | 175 | 177 | 240 | 248 | 323 | 339 | 142 | 146 |
| DL 16/10 | Invergowrie | *H. vulgare* | E | 258 | 262 | 246 | 262 | 170 | 174 | 175 | 177 | 240 | 248 | 323 | 339 | 142 | 146 |
| DL 16/13 | Invergowrie | *H. vulgare* | E | 258 | 262 | 246 | 262 | 170 | 174 | 175 | 177 | 240 | 248 | 323 | 339 | 142 | 146 |
| DL 16/15 | Invergowrie | *H. vulgare* | F | 258 | 262 | 246 | 256 | 170 | 174 | 175 | 177 | 240 | 248 | 323 | 339 | 142 | 146 |
| DL 16/16 | Invergowrie | *H. vulgare* | F | 258 | 262 | 246 | 256 | 170 | 174 | 175 | 177 | 240 | 248 | 323 | 339 | 142 | 146 |
| JB | Invergowrie | Unknown | G | 258 | 262 | 255 | 267 | 178 | 192 | 157 | 175 | 264 | 272 | 316 | 360 | 142 | 146 |

**Table S3** Thermocycling conditions for diagnostic PCR of aphid facultative endosymbionts and *APSE* amplification.

| Target | Time | Temperature (^o^C) | Repeat | Action |
| --- | --- | --- | --- | --- |
| 16S rDNA | 5 minutes | 95 | - | Initial denaturation |
|  | 30 s | 95 | 35 cycles | Denaturation |
|  | 45 s | 60 |  | Annealing |
|  | 45 s | 72 |  | Extension |
|  | 7 minutes | 72 | - | Final extension |
| 16S rDNA; *H. defensa., R. insecticola., S. symbiotica.* 16-23S rDNA | 2 minutes | 95 | - | Initial denaturation |
|  | 30 s | 95 | 35 cycles | Denaturation |
|  | 30 s | 55 |  | Annealing |
|  | 3 minutes | 72 |  | Extension |
|  | 7 minutes | 72 | - | Final extension |
| 16S rDNA; *PAXS, Rickettsiella sp.* | 2 minutes | 95 | - | Initial denaturation |
|  | 30 s | 95 | 35 cycles | Denaturation |
|  | 30 s | 55 |  | Annealing |
|  | 90 s | 72 |  | Extension |
|  | 5 minutes | 72 | - | Final extension |
| 16S rDNA *Rickettsia sp.,* Spiroplasma sp. | 2 minutes | 95 | - | Initial denaturation |
|  | 60 s | 95 | 35 cycles | Denaturation |
|  | 60 s | 55 |  | Annealing |
|  | 2 minutes | 72 |  | Extension |
|  | 5 minutes | 72 | - | Final extension |
| *APSE* P3, *APSE* P51 | 5 minutes | 95 | - | Initial denaturation |
|  | 30 s | 95 | 35 cycles | Denaturation |
|  | 45 s | 60 |  | Annealing |
|  | 45 s | 72 |  | Extension |
|  | 10 minutes | 72 | - | Final extension |
| *APSE* P35 | 5 minutes | 95 | - | Initial denaturation |
|  | 30 s | 95 | 35 cycles | Denaturation |
|  | 45 s | 65 |  | Annealing |
|  | 45 s | 72 |  | Extension |
|  | 10 minutes | 72 | - | Final extension |

**Table S4**: Best BLASTn hits for *APSE* endosymbiont marker sequences amplified from *R. padi* lines infected with *H. defensa* . Sequences were analysed for BLASTn similarity against sequences held on the NCBI database.

| **R. padi Asexual Line** | **Best BLASTn hit of Sequenced Data. *APSE* P35** | **Accession of Best BLASTn hit** | **% Identity/%Query Cover** |
| --- | --- | --- | --- |
| DL 16/03 | *Acyrthosiphon pisum* secondary endosymbiont phage 2 transfer protein (p35) gene, partial cds | EU02198.1 | 99/95 |
| DL 16/04 |  | EU02198.1 | 99/94 |
| DL 16/05 |  | EU02198.1 | 99/93 |
| DL 16/07 |  | EU02198.1 | 95/56 |
| DL 16/08 |  | EU02198.1 | 99/94 |
| DL 16/14 |  | EU02198.1 | 99/93 |
| **Best BLASTn hit of Sequenced Data. *APSE* P51** | | | |
| DL 16/03 | Bacteriophage APSE strain Lotus 74 hypothetical protein (P51) gene, partial cds | KT028666.1 | 98/96 |
| DL 16/04 |  | KT028666.1 | 98/96 |
| DL 16/05 |  | KT028666.1 | 96/98 |
| DL 16/07 |  | KT028666.1 | 98/90 |
| DL 16/08 |  | KT028666.1 | 98/91 |
| **Best BLASTn hit of Sequenced Data. *APSE* P3** | | | |
| DL 16/03 | Bacteriophage *APSE* strain S14-2 putative P-loop ATPase (P3) gene, partial cds | KC242512.1 | 97/93 |
| DL 16/04 |  | KC242512.1 | 98/89 |
| DL 16/05 |  | KC242512.1 | 97/93 |
| DL 16/07 |  | KC242512.1 | 96/93 |
| DL 16/08 |  | KC242512.1 | 96/95 |
